# Adenomatoid Tumor of Testis

**DOI:** 10.4137/cpath.s3091

**Published:** 2009-09-09

**Authors:** Waqas Amin, Anil V. Parwani

**Affiliations:** 1 Departments of Pathology and Biomedical Informatics, University of Pittsburgh School of Medicine, Pittsburgh, PA, USA

**Keywords:** adenomatoid tumor, paratesticluar masses

## Abstract

Adenomatoid tumors are responsible for 30% of all paratesticular masses. These are usually asymptomatic, slow growing masses. They are benign tumors comprising of cords and tubules of cuboidal to columnar cells with vacuolated cytoplasm and fibrous stroma. They are considered to be of mesothelial origin supported by histochemical studies and genetic analysis of Wilms tumor 1 gene expression. Excision biopsy is both diagnostic and therapeutic procedure. The main clinical consideration is accurate diagnosis preventing unnecessary orchiectomy. Diagnostic studies include serum tumor markers (negative alpha fetoprotein, beta HCG, LDH) ultrasonography (hypoechoic and homogenous appearance) and frozen section.

## Introduction

Adenomatoid tumors are rare and benign tumors of the male and female genital tract. They are also known by the pseudonym of “benign mesothelioma” of the intrascrotal tumors but they usually present as extra testicular masses, they are the most common paratesticular neoplasm and account for approximately 30% of all paratesticular masses.^1^,^2^ Occasionally they occur as an intratesticular mass. Although the histiogenesis of this rare neoplasm has been a source of controversy and it is now generally agreed that they are of mesothelial origin. Recent studies have provided evidence in favor of a mesothelial origin.^3^ The Wilms tumors 1 WT1 gene which is involved in normal growth and differentiation of mesothelial tissue, gonads, kidney and spleen has also been implicated in adenomatoid tumor development further supporting its mesothelial origin.^4^

## Clinical Features

Adenomatoid tumors show a predilection for white males they appear mostly in the third to fifth decades, mean age is 36 years.^2^,^5^ Adenomatoid tumor usually arises in male and female genital tract organs. In females they are found in uterus, fallopian tubes and ovarian hilus. In the male they occur in the epididymis, spermatic cord, prostate and ejaculatory duct. Mostly they arise within or around the lower or upper pole of the epididymis with equal frequency on both sides.^6^ Intratesticular adenomatoid tumor originates in the tunica albugines resulting in their peripheral location. They may also be found in the tunica vaginalis and rete testis.^2^ Adenomatoid tumors are the most common paratesticular neoplasms and account for approximately 30% of all paratesticular masses and unusual locations include the adrenal gland, pleura and lymph nodes resulting in diagnostic difficulties.^1^,^4^

They present either as an incidental finding or a slow growing scrotal mass. Enlargement is usually painless with normal scrotal skin and surrounding adenexa. Mostly they have been present asymptomatically for several years and are uniformly benign. Rarely it has presented with testicular pain and inflammation and also as an incidental finding in a chronic myeloid leukemia (CML) patient on imatinib therapy, but its implications for long term imatinib therapy are as yet undetermined.^7^ Adenomatoid tumors are usually small in size rarely exceeding 2 cmm^3^ (range is 0.5–5.0 cm). On ultrasonography they are typically appear as hyperechoic and homogeneous. Although they may also be hypoechoic.^2^

## Pathology

### Gross

On gross appearance these tumors are usually small, solid, firm, grayish white to tan and poorly to well circumscribed masses. They are situated between testicular tissue and tunica albugunia or lamina parietalis of tunica vaginalis testes. They can also appear as flattened and plaque like masses. On frozen section appearance shows atrophic testes with fibrous stromal proliferation.^8^

Extra genital adenomatoid tumors have been reported in ovaries and uterus. Multiple adenomatoid tumors have been reported in mesocolon and omentum, other reported locations include adrenal gland, liver, peritoneum, heart, pleura (mediastinal lymph node), appendix, pancreas. A comparison of adenomatoid tumors of sites other than testes is detailed in the Table 1.

### Histopathology

**Figure 1.**
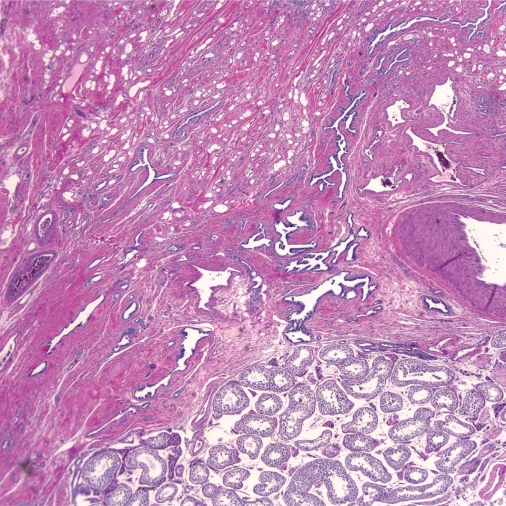
A low power view of a paratesticular adenomatoid tumor. Note the dilated tubules giving the appearance of endothelial spaces, adjacent to the normal uninvolved testicular parenchyma (4x, H & E).

**Figure 2.**
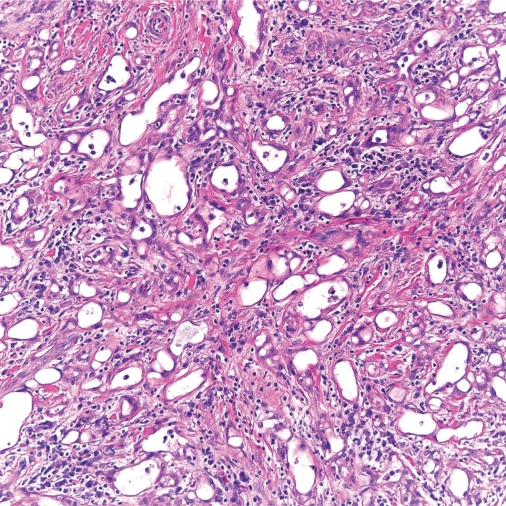
Higher power view showing the tubular pattern of growth with dilated spaces and intervening fibrous stroma with a smooth muscle component (20x H & E).

**Figure 3.**
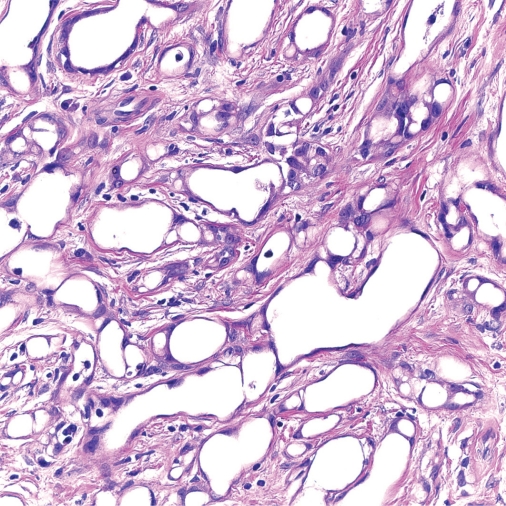
A higher power view of the neoplasm with predominant component consisting of tubules with intervening single cells with a vacuolated appearance, a characteristic finding in adenomatoid tumors (40x, H & E).

Adenomatoid tumors show a spectrum of histological patterns as adenoid or tubular glandular, angiomatoid, solid, cystic or transitional forms of all four above histological patterns.^4^ Microscopically, tumors appear as eosinophilic mesothelial cells in pattern of solid cords as well as dilated tubules that may look like endothelium in origin (Figs. 1 and 2). The pathognomic feature of the cells is a vacuolated cytoplasm with cytologic atypia (Fig. 3). Mitoses are typically not present. In most of the lesions, the stroma is fibrous but may occasionally contain a smooth muscle component (Fig. 2).^2^,^8^ This tumor is generally unencapsulated therefore their pattern of growth is uncharacteristic of benign neoplasms and frequently invade the surrounding tissue (Fig. 1).^15^ AT cells attain positively for Hyaluronidase sensitive muco-substance (HSM) with Alcian blue stain.^5^

### Immunohistochemistry

Immunohistochemical test show a presence of WT1 gene expression, calretinin (a 29-kilodalton, calcium binding protein is a specific marker for mesothelial cells and mesothelioma) and vimentin.^4^ They have also been reported to show positivity/reactivity for high and low molecular weight cytokeratin (CK), EMA and primary mesothelial monoclonal antibody HBME-1.^5^

Adenomatoid tumors show non-reactivity for epithelial/carcinoma markers CEA CD 15 (Leu M1) B72.3 MOC-31, Ber-ep4, LeA 135, factor VIII, CD34, Absence of staining for hemangioma (H) associated antigens factor VII and CD 34 excludes vascular tumors and mesenchymal lesions. Absence of inhibin expression helps exclude adrenal cortical and sex cord-stromal neoplasms.^16^

## Diagnosis

Ultrasonography reveals the nature of the lesion which is usually hypoechoic and solid. During ultrasonography a real time maneuver can be performed by the radiologist with the probe in one hand and pushing the testes downwards with a finger of the free hand. If the testes displace downwards and the mass remains at its site then it is concluded that the mass is paratesticular. This facilitates preoperative assessment and organ sparing surgery.

Absence of serum tumor markers like B-HCG, CEA, alpha-fetoprotein and LDH help to exclude malignant lesion. Histological evaluation of the specimen results in definitive diagnosis which is also supported by immunohistochemistry.

Magnetic Resonance Imaging (MRI) finding are helpful in pre-operatively determining if the palpable mass is originating from the albuginea of the testes or from the surrounding seminiferous tubules. Typically the scrotal MRI is executed using fast spin echo T2 weighted images in axial and sagittal planes. Unenenhanced and dynamic gadolinium-enhanced-gradient echo T1 weighted images are captured in axial plane and followed 30, 60 and 90 seconds after contrast administration and delayed gadolinium—enhances gradient echo T1-weighted images in sagittal and axial planes. A lens shaped mass is seen on unenhanced images originating from the testicular surface, the mass is somewhat hypointense in comparison with the testicular parenchyma. Dynamic and delayed enhanced images show thin uniform band of hypointense parenchyma around the mass showing early and persistent enhancement.^17^ Dynamic contrast enhanced MRI studies are helpful in differentiating testicular tumors from other testicular disorders.^18^

Abdominal CT is helpful in identifying extra genital adenomatoid tumors. Solitary hepatic adenomatoid tumor can be detected on routine abdominal ultrasound presenting as a hypervascular hyperechogenic tumor.^9^ For adenomatoid tumors arising in the adrenal gland MRI and CT are helpful in revealing the mass and detailing its extent and invasion.^11^

## Differantial Diagnosis

The differential diagnoses include adenocarcinoma of testes, hemangioma, malignant mesothelioma, yolk sac tumor, metastatic carcinoma of prostate, lymphoma and other testicular masses and tumors.

## Management and Prognosis

Excision biopsy is considered both diagnostic and therapeutic procedure. Adenomatoid tumors have not been known to ever recur or show malignant degeneration.^4^ The adequate use of tumor marker studies, utrasonography and intraoperative frozen sections can facilitate organ sparing surgery. The aim is to prevent unnecessary orchiectomy resulting in continuation of endogenous testosterone production and preserving fertility.^8^

## Conclusion

This rare, benign neoplasm provides a clinical diagnostic challenge with the aim to preserve endogenous testicular function. Recent research suggests its origin to be mesothelial even though these tumors occur in hormone responsive organ but no hormone receptors have been discovered in adenomatoid tumors and there is no proof that these are hormone sensitive.

## Figures and Tables

**Table 1.** Present radiological and pathological comparison of adenomatoid tumor of testis at other anatomic sites.

**Organ**	**Radiology**	**Gross**	**Microscopy**	**Immunohistochemistry**
**Liver** (cystic type)^9^	MRI: Hypointense on T1 weighted images and hyperintense on T2 weighted images. On Gd enhanced images it is hyperintense	Resembles hemangioma. Soft mass subcapsular hemorrhagic cut surface with microcystic structures non encapsulated	Cystic spaces of various sizes containing RBCs and colloid like matrix, lined by cuboidal to low columnar and flattened epithelioid cells surrounded by collagenous stroma. Epithelioid cells show small to large vacuoles, eosinophilic cytoplasm and round to oval nuclei. Fine collagenous bands constitute the Tumor border, no invasion into liver parenchyma	Positive for AE1/AE3 Cam 5.2 EMA CK7 CK19 (Consistent with mesothelial origin)
**Pancreas** (solid variant, usually cystic type)^10^	CT shows well circumscribed hypodense mass within the pancreas	Firm white circumscribed nodule	Mixed spindle cells and tubules lined by attenuated cuboidal cells	Positive for Keratin, vimentin, CK 5/6 EMA, CD 99, clretinin Negative for CD 34, Polyclonal carcinogenic embryonic antigen, bcl-2, smooth muscle actin, S 100, CD 117 and neuroendocrine markers (Consistent with mesothelial origin)
**Adrenal** (tubules and glands)^11^	CT: small mass in the adrenal gland MRI: Small mass with no invasion into the surrounding tissue	Multilocular cyst OR Well circumscribed nodule with solid cut surface	Interconnecting tubules and glands lined by plump epithelioid cells with plentiful eosinophilic cytoplasm to flat mesothelial like cell. Presence of adipose tissue, lymphoid aggregated and mucin producing areas	Positive for Calretinin, Cytokeratin 5/6 Negative for HMB45, CD35, myeloperoxidase (Consistent with mesothelial origin)
**Ovary^12^**	CT: Ovarian lesion with septa and small solid central portion	Solid with cystic component	Cuboidal to flat cells forming tubules and solid cords surrounded by patchy fibroid or myxoid stroma which may contain aggregates of inflammatory cells and smooth muscle fibers. Tubules may show small cystic dilations	Positive for Cytokeratin Negative for CD34 (Consistent with mesothelial origin)
**Uterus^13^**	MRI: Resembles leiomyoma without degeneration, shows low signal intensity with clear margins on T2 weighted and Gd- DTPA enhanced T1 weighted images. isointensity on T1 weighted images and patterns similar to normal myometrium in dynamic MRI	Solitary circumscribed nodular non encapsulated mass with smooth yellow to tan grey cut surfaces. May imitate areas of focal adenomyosis	Gland like spaces lined by a single layer of cuboidal cells with prominent nuclei. May displace fibromuscular stroma or infiltrate diffusely with scattered clusters cells	Positive for LMWK Cytokeratin, Cam 5.2, Calretinin, HMBE-1. (Consistent with mesothelial origin)
**Mesocolon and omentum^14^**	CT: Hypervascular tumor between uterus and rectum. Multiple small nodules in pelvic cavity	Well circumscribed gray tan. Elastic with small cystic spaces	Tubules and anastomosing channels lined by cuboidal or flattened cells with eosinophilic. Nuclei contain vesicles and are uniform oval to round	Positive for Pancytokeratin AE 1/AE 3, Vimentin Cytokeratin 5/6, Calretinin (Consistent with mesothelial origin)
